# A Novel Oncolytic Herpes Simplex Virus Design based on the Common Overexpression of microRNA-21 in Tumors

**DOI:** 10.13188/2381-3326.1000007

**Published:** 2018-10-18

**Authors:** M Marzulli, L Mazzacurati, M Zhang, WF Goins, ME Hatley, JC Glorioso, JB Cohen

**Affiliations:** 1 Department of Microbiology and Molecular Genetics, University of Pittsburgh School of Medicine, Pittsburgh; 2 Department of Oncology, St. Jude Children’s Research Hospital, USA

**Keywords:** Herpes simplex virus, Oncolytic viruses, miRNA-21, Dominant-negative U_L_9 gene

## Abstract

**Background:**

Recognition sequences for microRNAs (miRs) that are down-regulated in tumor cells have recently been used to render lytic viruses tumor-specific. Since different tumor types down-regulate different miRs, this strategy requires virus customization to the target tumor. We have explored a feature that is shared by many tumor types, the up-regulation of miR-21, as a means to generate an oncolytic herpes simplex virus (HSV) that is applicable to a broad range of cancers.

**Methods:**

We assembled an expression construct for a dominant-negative (dn) form of the essential HSV replication factor U_L_9 and inserted tandem copies of the miR-21 recognition sequence (T21) in the 3’ untranslated region. Bacterial Artificial Chromosome (BAC) recombineering was used to introduce the dnU_L_9 construct with or without T21 into the HSV genome. Virus was produced by transfection and replication was assessed in different tumor and control cell lines.

**Results:**

Virus production was conditional on the presence of the T21 sequence. The dnU_L_9-T21 virus replicated efficiently in tumor cell lines, less efficiently in cells that contained reduced miR-21 activity, and not at all in the absence of miR-21.

**Conclusion:**

miR-21-sensitive expression of a dominant-negative inhibitor of HSV replication allows preferential destruction of tumor cells in vitro. This observation provides a basis for further development of a widely applicable oncolytic HSV.

## Introduction

Oncolytic virus (OV) therapy is aimed at the selective destruction of cancer cells without harming healthy tissue [1]. Typically, OVs contain mutations that block lytic virus replication in normal cells but are complemented in cancer cells [2]. Oncolytic viruses derived from Herpes Simplex Virus Type 1 (oHSV) have shown efficacy in preclinical models of several types of cancer and safety in Phase I human trials, but therapeutic outcomes have been disappointing [3–5]. This is due in part to the attenuating mutations in these viruses that provide tumor selectivity, but also reduce lytic replication activity in tumors [6]. Thus there is a need for new strategies to protect normal cells in the tumor environment while supporting undiminished virus replication in the tumor cells. Recent studies have demonstrated that lytic virus replication can be brought under the control of cellular microRNAs that are differentially expressed in normal and cancer cells [7–9].

Micro (mi)RNAs are non-coding, 22-23 nucleotides long, uncapped RNAs that are processed from longer precursor transcripts [10]. They negatively regulate gene expression by binding to complementary mRNA targets, and dysregulated miRNAs can cause aberrant cell phenotypes, including cancer [11]. miRNA expression profiles of cancer cells typically show both up- and down-regulated miRNAs, but each cancer type has a unique miRNA signature showing dysregulation of different sets of miRNAs [12–14]. Thus targeted therapies based on these unique signatures are applicable only to the corresponding cancer type.

Lytic HSV replication is controlled by 2 essential immediate early (IE) genes, ICP4 and ICP27. In the absence of either gene product, the virus fails to initiate the lytic gene expression cascade required for replication. Accordingly, miRNAs that are specifically down-regulated in cancer cells can be taken advantage of to block virus replication in normal cells while allowing replication in cancer cells by engineering one of the essential viral genes for recognition of its mRNA by one or more of these specific miRNAs [7–9]. However, since there is little overlap in the miRNAs that are down-regulated in different cancer types, no single cancer down-regulated miRNA can provide proper control of oncolytic virus replication across cancer types.

Remarkably, studies of a wide range of cancer types have found that one miRNA, the oncomir miR-21, is up-regulated nearly universally in cancer cells [15]. This recognition and functional studies are consistent with a key role for miR-21 in the development and/or maintenance of the neoplastic state. Using a tissue-targeted, doxycycline-sensitive miR-21 transgenic mouse strain, Medina and co-workers observed that miR-21 over expression in hematopoietic tissues resulted in extensive clonal expansion of invasive cell populations that formed solid tumors on transplant into nude mice [16]. The authors in addition showed that doxycycline-mediated silencing of the mir-21 transgene caused universal reversal of the neoplastic state, including rapid regression of transplant tumors, thus demonstrating a complete correlation between miR-21 over expression and both tumor formation and maintenance. In other studies, miR-21-null mice showed a significant reduction in papilloma formation compared with wild type mice in a chemically-induced skin cancer model and presented significantly fewer tumors on the lungs than mir-21^+/+^ mice in a K-ras-induced lung cancer model [17,18]. Consistent with these and related findings both in vitro and in vivo [19–23], a variety of predicted or experimentally validated miR-21 targets are involved in cell cycle regulation, apoptosis, cell migration and stem cell self-renewal [24–29].

**Figure 1:**
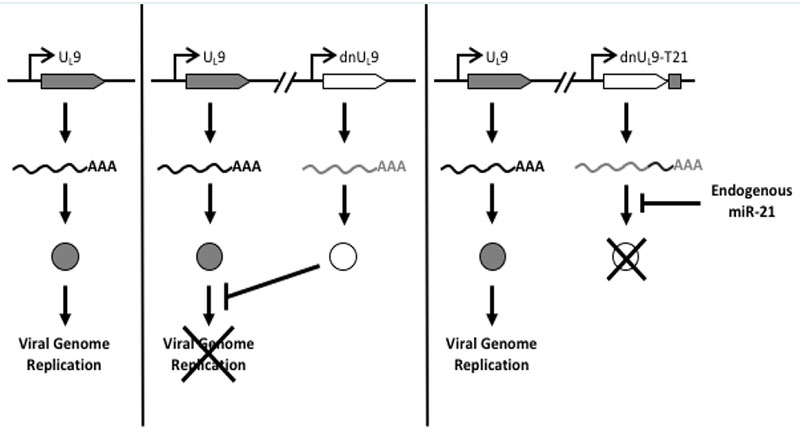
Design of miR-21 dependent HSV replication. Left, translation of mRNA transcribed from the U_L_9 gene produces a protein that is required for HSV replication; center, dnU_L_9 protein expressed from the same viral genome blocks Ori binding by U_L_9 protein to limit replication; right, miR-21 binding sites inserted into the 3’UTR of the dnU_L_9 gene enable miR-21 binding to the cognate mRNA to prevent dnU_L_9 protein-mediated interference with viral genome replication.

We have sought to take advantage of the common up-regulation of miR-21 in cancer cells by creating a novel control circuit in which the host cell miRNA induces, rather than represses, lytic HSV replication. Upon HSV entry into the cell, the lytic life cycle progresses from IE gene expression to expression of the early (E) genes involved in viral genome replication. U_L_9 is an early gene encoding an 851 amino-acid (aa) protein that functions in the initiation of viral DNA synthesis [30]. The U_L_9 protein [Origin (Ori) Binding Protein or OBP] has a C-terminal DNA binding domain (aa 535-851) that specifically recognizes the viral origins of replication [31,32]. The N-terminal region mediates protein dimerization and possesses DNA-dependent ATPase and 3’-5’ helicase activities [32–36]. The U_L_9 protein interacts physically with a number of the other viral proteins required for Ori-dependent DNA replication [37–39]. A dominant-negative version of U_L_9, U_L_9-C535C consisting of the C-terminal DNA binding domain, has been shown to inhibit HSV genome replication by occluding the Ori binding sites of U_L_9 [40–44]. We reasoned that incorporation of a miR-21-responsive U_L_9-C535C (“dnU_L_9”) construct into the HSV genome would allow unimpaired virus replication in miR-21-overexpressing cancer cells while blocking replication in normal cells in the tumor environment (Figure 1). Thus the goal of this work was to create a safe and effective oHSV backbone that provides a general level of safety and efficacy for the treatment of cancer.

## Materials and Methods

### Cell culture

Human osteosarcoma U2OS, epidermoid carcinoma A431, lung adenocarcinoma A549, and African green monkey kidney Vero cells were from ATCC (Manassas, VA) and were grown in a 5% CO_2_ incubator at 37 °C in ATCC-recommended medium supplemented with 5-10% (v/v) fetal bovine serum (Sigma, St. Louis, MO). U2OS-ICP4-Cre cells, generated by retroviral transduction of U2OS-ICP4 cells essentially as described [45], were kindly provided by Y. Miyagawa (University of Pittsburgh). Primary miR-21^+/+^ and miR-21^−/−^ Mouse Embryonic Fibroblasts (MEFs) were as described [18].

### Plasmids

pfLuc-T21 contains four tandem repeats of the reverse complement of the hsa-miR-21 sequence separated by 8-nucleotide spacers (T21), while pfLuc-Tcon contains four tandem repeats of the hsa-miR-21 reverse sequence in the same configuration (Tcon). Both plasmids were constructed by insertion of annealed complementary oligonucleotides into the 3’UTR of the firefly luciferase (fLuc) gene in pMIR-REPORT” (miRNA Expression Reporter Vector System; Ambion, Austin, TX). Oligonucleotides were T21F, T21R, TconF, and TconR (Table 1). Annealed oligonucleotides were digested with SpeI and SacI, and ligated to SpeI-SacI-digested pMIR-REPORT^™^.

### Luciferase Assay

U2OS and Vero cells were co-transfected with a renilla luciferase expression plasmid (prLuc) and different combinations of pfLuc-T21 or pfLuc-Tcon and miR-21 or anti-miR-21 miRNA Precursors (Ambion). Relative expression levels were measured the following day, essentially as described [9].

### HSV genome engineering

The U_L_9-C535C coding sequence and 3’UTR were amplified from 3L^BAC^ DNA (see below) with primers C535C-F and C535C-R (Table 1). A fragment containing the RSV promoter and SV40 intron/LacO operator was isolated from pOPI3CAT (Lac Switch II Inducible Mammalian Expression System, Stratagene, La Jolla, CA) and inserted in front of the U_L_9-C535C sequence. The SV40 early polyadenylation (polyA) region was then inserted directly 5’ to the promoter and the T21 element from pfLuc-T21 was introduced into the NotI site in the U_L_9-C535C 3’UTR. The polyA-dnU_L_9 cassettes without or with T21 were then introduced into 3L^BAC^ in the 3’ UTR of the U_L_53 gene using the double Red recombination procedure of Tischer, et al. [46], creating vector constructs 3Ldn9^BAC^ and 3Ldn9T21^BAC^, respectively. 3L^BAC^ contains the wild-type glycoprotein B (gB) gene but is otherwise identical to KG^BAC^ described by Mazzacurati, et al. [9]. Changes were verified by PCR analysis, FIGE analysis of restriction enzyme digests, and sequencing through the inserted DNA.

### Virus production, growth curves and plaque sizes

BAC DNAs were converted to infectious viruses by transfection of U2OS-ICP4-Cre cells, essentially as previously described [9]. Amplification of isolated plaques and confirmation of accurate loss of the BAC/lacZ region were also as described [9]. Virus growth curves were established after infection of confluent monolayer cells at 1 genome copy (gc) per cell. Supernatants were collected from triplicate wells for each time point and their gc content was determined by real-time quantitative (q) PCR (see below). The results were plotted as fold increase over input. Plaque sizes were measured after infection of confluent monolayer cells at very low Multiplicity of Infection (MOI) and the addition of high-density media (1% v/v methylcellulose in DMEM/10% FBS) at 1 hour post-infection (hpi) to block secondary infection. At each time point, pictures were taken of the same plaques and the plaque areas were measured.

### qPCR for viral genomes

Viral genome copy titers were determined by quantitative PCR for the viral gD gene, as described [9].

### qRT-PCR for miR-21 levels

RNA extraction procedures were as described [9]. Mature hsa-miR-21 levels in confluent U2OS, A431, A549, and Vero cells were separately determined relative to RNU43 and RNU6B according to the TaqMan Small RNA Assays Protocol (Applied Biosystems/Life Technologies, Carlsbad, CA). TaqMan primers and probes were from Applied Biosystems (Table 1). All TaqMan PCR reactions were performed in triplicate.

### Statistical analyses

Unpaired t-tests were performed using GraphPad QuickCalcs (http://www.graphpad.com/quickcalcs/ConfInterval1.cfm; November 2014). The natural logarithms of genome copy numbers from virus growth curves were used for linear regression analysis by GraphPad Prism version 6.04 for Windows (GraphPad Software, La Jolla, CA), as recommended for statistical comparison of viral growth curves by Wang and Bushman [47].

## Results

### Validation of a miR-21 response element

**Figure 2:**
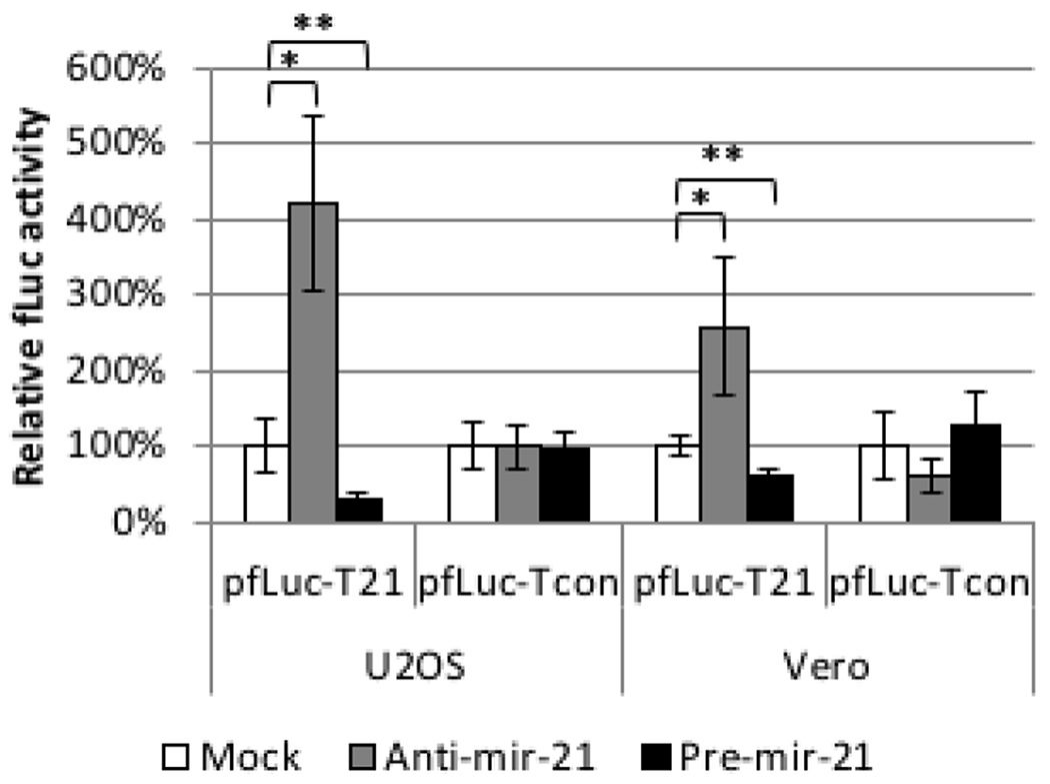
Functionality of the T21 element. U2OS and Vero cells were co-transfected in triplicate with pfLuc-T21 or pfLuc-Tcon, synthetic Anti-miR-21 or Pre-miR-21, and prLuc. Cell extracts prepared 24 h later were assayed for fLuc and rLuc activity. fLuc values were normalized to rLuc values and are represented as % activity relative to no-miRNA (Mock) controls (100%). * *p*<0.01; ** *p*<0.05 (unpaired t-test).

We used a luciferase assay to test the functionality of a miR-21 response element (T21) consisting of four tandem copies of the reverse complement of mature miR-21 separated by different 8-nucleotide spacers. As a control, we created a similar element incorporating four tandem copies of the reverse sequence of mature miR-21 (Tcon). We inserted Tcon and T21 into the 3’UTR of a firefly luciferase (fLuc) expression plasmid (pfLuc-Tcon and pfLuc-T21, respectively) and performed co-transfection experiments with a Pre-miR-21 or Anti-miR-21 RNA on human U2OS and monkey Vero cells; human and monkey miR-21 are identical in sequence [48]. Co-transfections were performed in the presence of a renilla luciferase (rLuc) expression plasmid (prLuc) for normalization. Figure 2 shows that pfLuc-T21 co-transfection with Pre-miR-21 significantly reduced fLuc activity in both cell lines at 24 h compared with mock co-transfection, while co-transfection with Anti-miR-21 resulted in significantly increased fLuc activity. In contrast, the same co-transfections with pfLuc-Tcon revealed no significant differences in either U2OS or Vero cells. These results demonstrated that the T21 sequence, unlike the Tcon sequence, is responsive to miR-21.

### Engineering and miR-21-dependent viability of dnUL9 recombinant viruses

**Figure 3:**
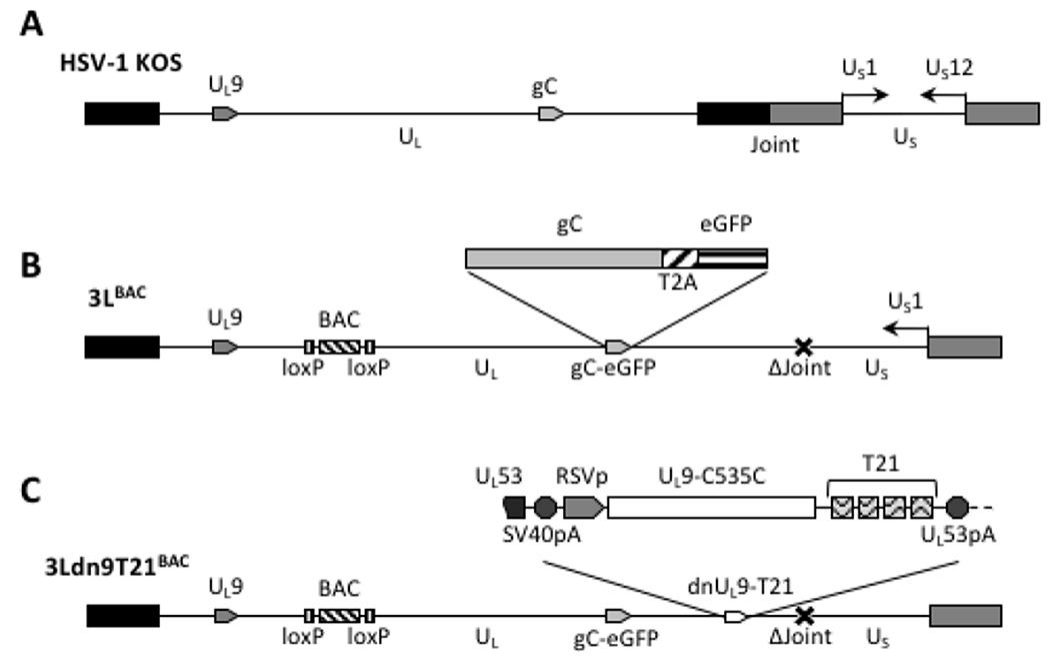
Structures of the HSV-1 genome and engineered BAC constructs. **(A)** Schematic representation of the linear strain KOS genome. U_L_, unique long segment; U_S_, unique short segment; joint, internal repeat region separating U_L_ and U_S_. Boxes, inverted repeats flanking U_L_ (black) and U_S_ (grey). The relative positions of the U_L_9 and gC genes and the orientation of the U_S_1 (ICP22) and U_S_ 12 (ICP47) genes (prototype isomer [69]) are indicated. **(B)** Linear representation of the 3L^BAC^ genome. The illustration includes the BAC/lacZ region between loxP sites in the U_L_37/U_L_38 intergenic region, the C-terminal extension of the gC ORF with a T2A-eGFP fusion, and the deletion (Δ) of the entire joint. Note that the U_S_ segment is in the reverse reverse orientation relative to U_L_ such that U_S_ 1 is transcribed while the joint deletion eliminates the U_S_ 12 promoter. **(C)** The 3Ldn9T21^BAC^ genome illustrating the position and structure of the miR-21-sensitive dnUL9 expression cassette inserted into the 3L^BAC^ backbone. Functional elements of the insertion between the U_L_53 ORF and polyA (pA) signal include the SV40 polyA region, the RSV promoter (RSVp), the dnU_L_9 ORF (U_L_9-C535C), and the repeat copies of the miR-21 recognition sequence (T21).

We used double Red recombination in E. coli to introduce a dnU_L_9 expression construct without (dn9) or with (dn9T21) the T21 element from pfLuc-T21 into the 3’UTR of the U_L_53 gene of 3L^BAC^, a bacterial artificial chromosome (BAC) containing a modified version of the HSV-1 strain KOS genome (Figure 3) [46]. The insertion separated the U_L_53 gene from its polyadenylation (polyA) signal and we therefore included the SV40 early polyA region directly upstream of the dnU_L_9 cassette to allow proper processing of the U_L_53 primary transcript (Figure 3C). The Rous Sarcoma Virus (RSV) promoter was used to direct dnU_L_9 transcription in order to achieve expression prior to the onset of transcription of the resident early U_L_9 gene. Like KG^BAC^ described previously [9], 3L^BAC^ is deleted for the internal repeat (joint) region separating the unique long (U_L_) and short (U_S_) segments of the HSV genome, the open reading frame (ORF) of glycoprotein C (U_L_44), a late gene, is fused in frame via a 2A peptide sequence to the enhanced green fluorescent protein (eGFP) ORF to allow visual monitoring of post-replication viral gene expression [49,50], and the BAC elements, including a LacZ expression cassette, are straddled by loxP sites for removal by Cre recombinase (Figure 3B); 3L^BAC^ differs from KG^BAC^ by the absence of mutations in the gB gene. Insertion of the dn9 and dn9T21 cassettes produced recombinants 3Ldn9^BAC^ and 3Ldn9T21^BAC^, respectively. Isolates were confirmed by FIGE analysis of restriction enzyme digests along with PCR and sequencing across the insertion boundaries.

Two confirmed isolates of each recombinant and 3L^BAC^ as a control were transected into a Cre-expressing U2OS cell line (U2OS-ICP4-Cre). We observed plaque formation by both of the 3L^BAC^ and both of the 3Ldn9T21^BAC^ isolates, but not by either 3Ldn9^BAC^ isolate. This initial observation was consistent with the interpretation that dnU_L_9 expression blocked virus replication and that in U2OS cells this block was reversed by the miR-21-responsive element in the dnU_L_9T21 gene.

### Cell specificity of dnU_L_9T21 virus growth

**Figure 4:**
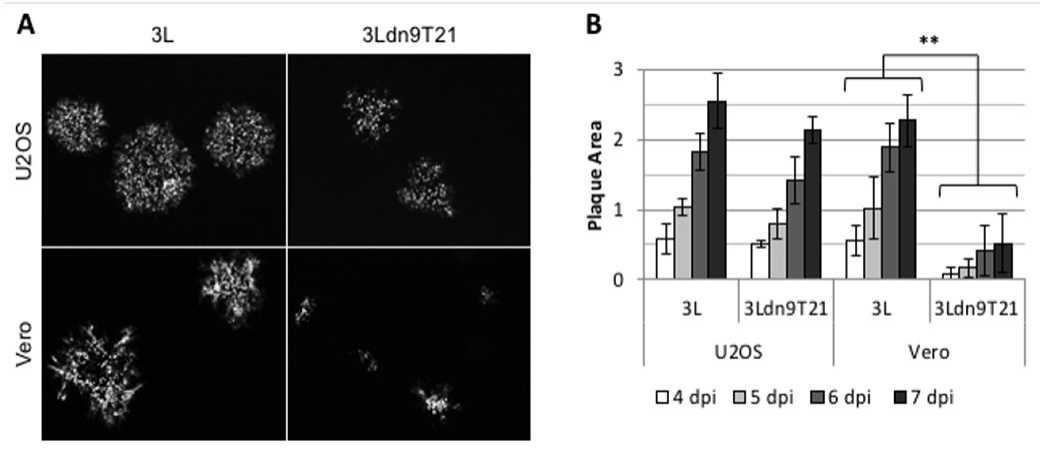
Comparison of 3L and 3Ldn9T21 plaques on U2OS and Vero cells. **(A)** Plaques visualized by GFP fluorescence at 5 dpi. **(B)** Average plaque sizes ± SD measured at 4-7 dpi. ** 3L plaques were significantly larger than 3Ldn9T21 plaques on all 4 days (*p*<0.05).

We isolated and amplified individual plaques from 3L^BAC^ and 3Ldn9T21^BAC^ transfections of U2OS-ICP4-Cre cells and used X-gal staining to identify loxP recombinants; accurate Cre-mediated removal of the BAC-lacZ region between the loxP sites was confirmed by DNA sequencing. One BAC-deleted virus isolate from each transfection, referred to as 3L and 3Ldn9T21, respectively, was further amplified and the biological titers of the resulting stocks on Vero cells and three established human tumor lines representing different tumor types were determined by plaque assay. In addition, we determined the physical titers [in genome copies (gc)/ml] of the two stocks by real-time quantitative (q) PCR for the viral gD (U_S_6) gene [9]. The results in Table 2 show that on each cell line, the difference between the two viruses in the number of input viral genomes required to produce a plaque (gc/pfu ratio) was less than 2-fold, indicating comparable entry efficiencies. However, while the 3L and 3Ldn9T21 plaques were similar in size on each of the three human cell lines, on Vero cells their sizes were dramatically different (Figure 4A). We measured the plaque sizes of both viruses on U2OS and Vero cells on days 4-7 post infection (dpi) and found that the 3L plaques on Vero cells were on average 4.4 fold larger than the 3Ldn9T21 plaques while the average difference on U2OS cells was only 1.2 fold (Figure 4B). This observation suggested that Vero cells may contain less functional miR-21 activity than U2OS cells, allowing higher dnU_L_9 expression resulting in reduced virus replication and spread to neighboring cells. It is likely that the relatively high gc/pfu value for 3Ldn9T21 on Vero cells shown in Table 2 can be explained at least in part by the reduced counting accuracy of small plaques.

**Figure 5:**
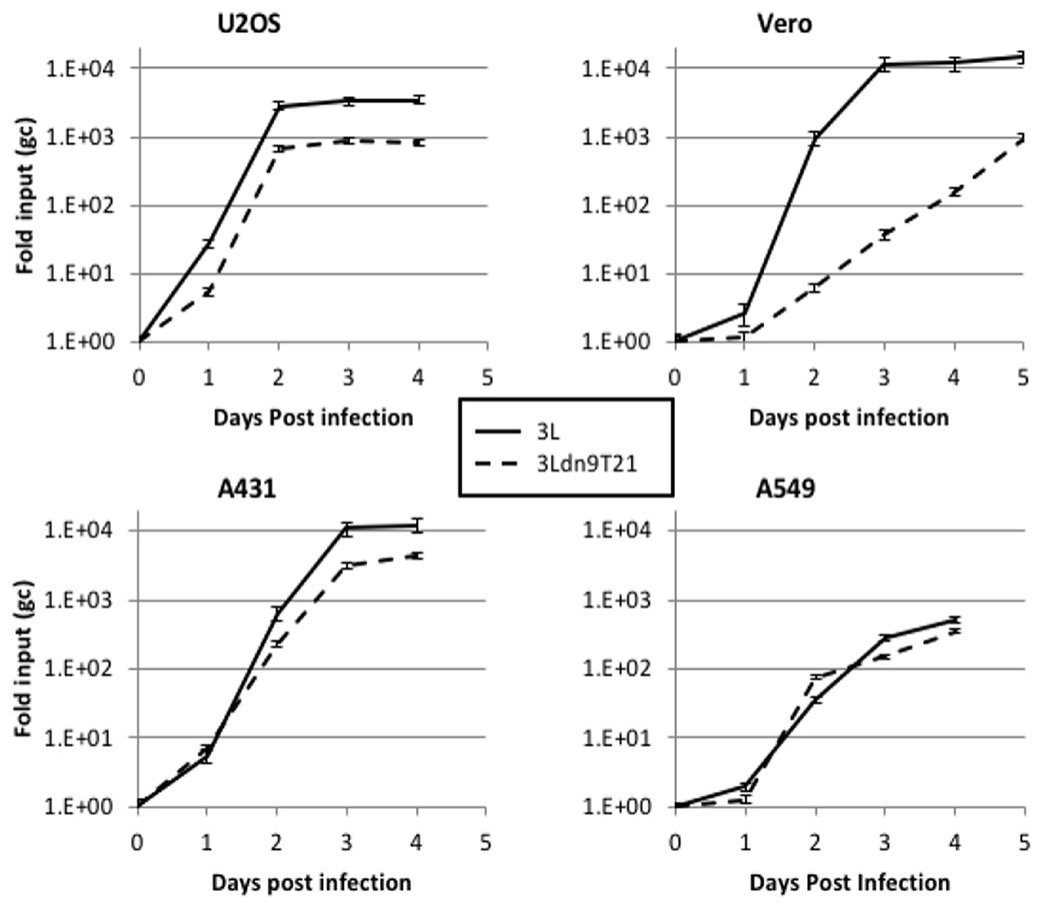
Comparison of 3L and 3Ldn9T21 growth kinetics on Vero cells (*p*<0.0001) and human tumor lines. Cells were infected with 3L or 3Ldn9T21 at 1 gc/cell and total gc in the media of triplicate wells were measured at 1-5 dpi. Data is plotted as fold increase over input and statistical comparisons were performed by the method of Wang and Bushman [47].

To strengthen the correlation between miR-21 activity and 3Ldn9T21 replication, we examined the growth kinetics of 3L and 3Ldn9T21 on the 3 human tumor cell lines and Vero cells. Infections were performed at 1 gc/cell, total gc content in the media was determined at 1–5 dpi, and the fold increase over input was plotted as a function of time (Figure 5). While no significant differences in growth kinetics were observed between the two viruses on the 3 human tumor lines, 3Ldn9T21 growth on Vero cells was substantially slower than that of 3L. At 3 dpi, the yields of 3Ldn9T21 differed by less than 5-fold from those of 3L on U2OS, A431 and A549 cells, whereas the difference on Vero cells was approximately 300-fold. 3L growth on Vero cells reached a plateau at 3 dpi while slow 3Ldn9T21 growth continued at 5 dpi. These results supported the conclusion that the three human tumor lines contained enough functional miR-21 to block abundant dnU_L_9 production from 3Ldn9T21, thus allowing replication similar to 3L, while Vero cells lacked sufficient miR-21 activity for effective repression of the inhibitory gene function.

### Virus growth in miR-21 knock-out cells

**Figure 6:**
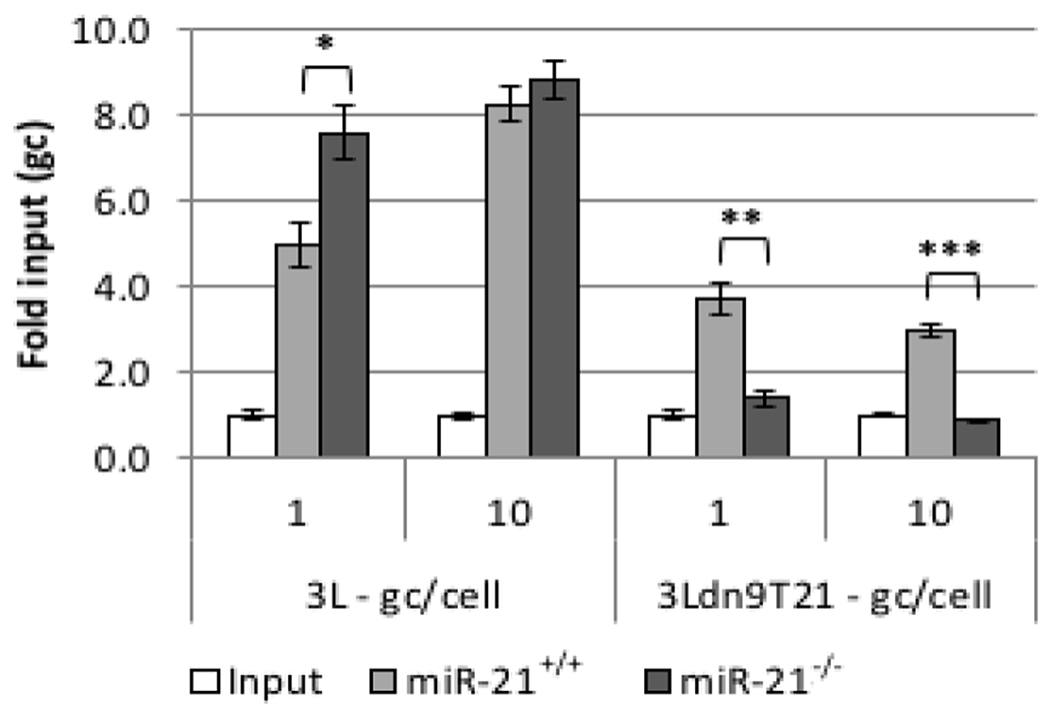
Virus production by wild-type and miR-21 knock-out MEFs. Cells were infected with 3L or 3Ldn9T21 at 1 or 10 gc/cell and total gc in the media of triplicate wells was determined at 24 hpi. Data is shown as fold increase over input. * *p*<0.01; ** *p*<0.001; *** *p*<0.0001 (unpaired t-test).

Surprisingly, quantitative RT-PCR showed that the level of miR-21 was approximately 1.5–2 times higher in Vero cells than in U2OS and A549 cells but ~30-fold lower than in A431 cells when normalized to endogenous RNU43 levels (data not shown). However, it is unlikely that RNU43 levels are the same between these entirely unrelated cells lines such that the results of these types of comparisons can be misleading [51]; other endogenous normalization standards suffer from the same uncertainty. Therefore, to confirm the miR-21 dependence of 3Ldn9T21 virus growth, we used primary cultures of mouse embryonic fibroblasts (MEFs) isolated from wild-type (miR-21^+/+^) and miR-21 knock-out (miR-21^−/−^) mice [18,52]. We infected the cells at 1 or 10 gc/cell and determined the genome titers in the media at 24 hpi. As shown in Figure 6, whereas 3L yields were comparable between the two cell lines, 3Ldn9T21 production was significantly impaired on the miR-21^−/−^ MEFs compared to the miR-21^+/+^ MEFs. These results supported the conclusion that 3Ldn9T21 replication is blocked in the absence of miR-21

## Discussion

Suitable oncolytic viruses combine the often conflicting properties of high replication efficiency in and selectivity for tumor cells. HSV selectivity for cancer cells has been accomplished by deletion of one or more viral genes, such as the γ_1_34.5 neurovirulence gene [53], the U_L_39 viral ribonucleotide reductase (ICP6) gene [54], the U_S_3 protein kinase gene [55,56], and/or the U_s_12 gene [57] whose product, ICP47, blocks MHC class I antigen presentation [58]. However, while representatives of this type of oHSV have shown excellent safety profiles in early-phase clinical studies, evidence of efficacy has remained anecdotal. This is likely due, at least in part, to incomplete complementation of the deleted viral function(s) in tumor cells, resulting in reduced replication efficiency. Accordingly, considerable effort has been directed in recent years toward the development of oHSVs that conditionally express the complete range of viral lytic functions. While several different strategies have shown promising results in animal models, the modifications designed to ensure the tumor selectivity of these viruses are invariably tailored to a specific tumor type. For example, expression of the γ_1_34.5 gene under control of the nestin enhancer dramatically increased HSV replication in glioblastoma cell lines and primary glioma cells without increasing replication in astrocytes [6], but the nestin enhancer is highly active only in tumors of the nervous system. Thus, while the general strategy of transcriptional targeting is applicable to other tumors [59], the implementing control elements will be different depending on the tumor type. We and others have shown that tumor-specific HSV replication can also be achieved by the incorporation into essential genes of response sequences for miRNAs that are down-regulated in the target tumor compared to its environment [7–9]. However, different sets of miRNAs are down-regulated in different tumors and thus this post-transcriptional targeting strategy also requires vector tailoring by tumor type [12–14]. A third method to limit lytic HSV replication to tumor cells is transductional retargeting involving disruption of the normal receptor recognition elements of the viral envelope (detargeting) and insertion of heterologous ligands for tumor-associated receptors to render virus entry dependent on recognition of the targeted receptor [60–62]. While certain receptors may be over-expressed in more than one tumor type, none are over-expressed as broadly in tumors as miR-21. Thus we sought to develop an oHSV backbone that would be dependent on miR-21 for replication to provide general protection of normal cells in the tumor environment without diminishing replication in a wide range of tumor cells. Our approach was thereby intended to combine the favorable aspects of traditional and newer oHSVs without the downsides of defective genes causing attenuation or the restricted applicability of a tumor-tailored virus. Importantly, accumulating evidence indicates that loss of miR-21 expression diminishes the neoplastic phenotype [16,21,23], suggesting that heterogeneity in intratumoral miR-21 levels will at worst spare only the least malignant cells.

Our study presents evidence that a miR-21-sensitive dominant inhibitor gene engineered into the HSV genome can limit virus replication in cells that express little or no active miR-21 while allowing vigorous replication in different types of tumor cells. Since miR-21 is associated with cell proliferation, we faced the difficulty of finding non-transformed cells that would grow in culture yet not express significant miR-21 to test for differential virus replication in normal and tumor cells in vitro. Although Vero cells are transformed and grow rapidly in culture, the results of our 3Ldn9T21 growth experiments led us to believe that these cells express less functional miR-21 than the 3 human tumor cell lines used in this study, U2OS, A431 and A549. However, when we measured the miR-21 levels in these cell lines relative to RNU43, the results did not correlate with the virus growth data. Instead, we used cultures of miR-21 knock-out MEFs to examine 3Ldn9T21 replication in the guaranteed absence of miR-21, and compared these host cells to matched wild-type MEFs to eliminate cell-type differences. The results demonstrated that 3Ldn9T21 was unable to replicate in the absence of miR-21 while it replicated to similar levels as the control 3L virus in the wild-type cells.

Our unexpected result from quantitative RT-PCR analyses that Vero cells appear to express more miR-21 than U2OS and A549 cells likely reflects the absence of reliable standards for normalization of miRNA levels between cells of diverse origin [51]. Our replication and luciferase data suggested that Vero cells do express a certain amount of miR-21, but 3Ldn9T21 grows slower in these cells than 3L while the two viruses grow at the same rate in U2OS, A431 and A549 cells, indicating that these human tumor lines express more functional miR-21 than Vero. We have performed qRT-PCR normalization to standards other than RNU43, such as RNU6B, and observed that the results vary prominently as a function of the standard. Thus we feel that functional assays like the ones used in this study offer a more reliable read-out of the activity of a specific miRNA than physical methods like qRT-PCR, Northern blots and RNA-Seq. Implicit in this suggestion is the possibility that only a portion of the specific miRNA is functional in a given cell.

While our study suggests proof of concept, our current vector is not optimized for the necessary studies of safety and efficacy in animal models. First, the KOS strain of HSV is less virulent than certain other laboratory strains and clinical isolates and thus oncolytic vectors derived from the KOS strain may not be optimally effective [63,64]. However, more aggressive vectors will require stronger safety features and it will be of interest to determine if our dn9T21 system will be adequate in these situations. Second, although in our experience the 3Ldn9T21 virus can be grown on miR-21-over-expressing tumor cells without selection of dnU_L_9 loss-of-function mutants (unpublished results), the emergence of such mutants in vivo as a result of tumor cell heterogeneity (varying levels of miR-21) can not a priori be excluded. In an unrelated study, we have found that the frequency of HSV transgene inactivation due to selective pressure can be dramatically reduced by insertion of a second copy of the transgene into the viral genome in such a manner that recombination between the 2 copies results in the formation of defective genomes [65]. Third, it has been reported that deletion of the joint diminishes virus replication in vivo [66], implying that restoration of the joint should be advisable although this would reduce the available space for insertion of transgenes that may enhance therapeutic efficacy. It remains to be determined, however, whether the joint deletion affects our vector in the same manner. In the published study [66], the in vivo replication deficit may have been caused by the loss of the joint-based ICP22 (U_s_1) promoter while in the genome isomer used to produce our vector (Figure 3A), the ICP47 (U_s_12) gene is located adjacent to the joint and the ICP22 gene is controlled by the remaining intact copy of the promoter in the U_s_ terminal repeat. As a result, the ICP47 gene is not expressed from our vector, which may facilitate immune recognition and clearance of infected tumors in vivo [57], while the replication-stimulatory function of ICP22 is preserved. Future studies will explore this issue and examine the benefits of using a more aggressive HSV strain and duplicate copies of the dn9T21 expression cassette as we move to evaluate our conditional replication control system in vivo.

Consistent with the evidence that miR-21 is expressed in proliferating cells [19,22,67], including unmodified MEFs [18,68], we found that 3Ldn9T21 replicates as efficiently as 3L in wild-type MEFs, indicating that the dn9T21 system will leave certain normal cells unprotected. In adults, few organs contain a significant population of dividing cells and thus inoculation of the virus directly into solid tumors may have no deleterious effects. However, should evidence of off-target replication arise, tailored safety measures referred to earlier can be added to protect specific cells. Indeed, while our approach may provide a global strategy to limit lytic HSV replication to rapidly dividing cells, we recognize that certain applications may require additional modifications to the viral backbone. Once optimized, however, we anticipate that our platform will be suitable for the treatment of a wide variety of tumors without extensive backbone re-engineering.

## Figures and Tables

**Table 1.** Oligonucleotides used in this study

Name	F(orward)	R(everse)	Description
T21(pfLuc-T21)	aaaaa*ACTAGT***GCGGCCGC**gtctcgg gaccgcactcgttTCAACATCAGTCTGA TAAGCTAtagtaccagTCAACATCAGT CTGATAAGCTAaggatcctTCAACAT CAGTCTGATAAGCTAatgactgcTCA ACATCAGTCTGATAAGCTA***ctcgag*** ctcaaaaa	tttttgag***ctcgag***TAGCTTATCAGACTG ATGTTGAgcagtcatTAGCTTATCAG ACTGATGTTGAaggatcctTAGCTTAT CAGACTGATGTTGActggtactaTAGC TTATCAGACTGATGTTGAaacgagtg cggtcccgagac**GCGGCCGC***ACTAGT*t tttt	Underlined, hsa-miR-21 target repeats; uppercase italics, Spel site; uppercase bold, NotI site; lowercase bold italics, Xhol site; shaded, SacI site
Tcon(pfLuc-Tcon)	aaaaa*ACTAGT***GCGGCCGC**gtctcgg gaccgcactcgttATCGAATAGTCTGAC TACAACTtagtaccagATCGAATAGTC TGACTACAACTaggatcctATCGAAT AGTCTGACTACAACTatgactgcATC GAATAGTCTGACTACAACT***ctcgag*** ctcaaaaa	tttttgag***ctcgag***AGTTGTAGTCAGACT ATTCGATgcagtcatAGTTGTAGTCA GACTATTCGATaggatcctAGTTGTA GTCAGACTATTCGATctggtactaAGT TGTAGTCAGACTATTCGATaacgagt gcggtcccgagac**GCGGCCGC***ACTAG* *T*ttttt	Underlined, control target repeats; uppercase italics, Spel site; uppercase bold, NotI site; lowercase bold italics, Xhol site; shaded, SacI site
C535C	aaaaaa*ccggt*GCCACCATGgatcccga ggcgtcgctg	aaaaaaaaaaa**gcggccgc**TTAtagggtgc taaagttcac	Uppercase, Kozak consensus sequence; underlined, stop codon; italics, Agel site; bold, NotI site
hsa-miR-21 (qRT-PCR)	TaqMan MicroRNA Assays Cat.# 4427975 ID: 000397^a^		
RNU6B (qRT-PCR)	TaqMan MicroRNA Assays Cat.# 4427975 ID: 001093^a^		
RNU43(qRT-PCR)	TaqMan MicroRNA Assays Cat.# 4427975 ID:001095^a^		

a Applied Biosystems

**Table 2.** Number of gc required to produce a single plaque on different cell lines (gc/pfu)

HSV	gc/ml^a^	U2OS^b^	Vero^b^	A431^b^	A549^b^
3L	6.50×10^10^	1.15×10^9^ pfu/ml	2.35×10^8^ pfu/ml	2.60×10^8^ pfu/ml	3.00×10^8^ pfu/ml
		**56.5 gc/pfu**	**276.6 gc/pfu**	**250.0 gc/pfu**	**216.7 gc/pfu**
3Ldn9T21	1.32×10^10^	1.80×10^8^ pfu/ml	2.45×10^7^ pfu/ml	4.70×10^7^ pfu/ml	7.80×10^7^ pfu/ml
		**73.3 gc/pfu**	**538.8 gc/pfu**	**280.8 gc/pfu**	**169.2 gc/pfu**

a Physical titer of input virus

b Biological titer (upper) and gc:pfu ratio (lower, bold)

## Abbreviations

HSV: Herpes Simplex Virus
miR: microRNA/miRNA
dn: Dominant Negative
BAC: Bacterial Artificial Chromosome
oHSV: Oncolytic HSV
IE: Immediate Early
MEF: Mouse Embryonic Fibroblast
FIGE: Field Inversion Gel Electrophoresis
gc: Viral Genome Copies
pfu: Plaque Forming Unit
MOI: Multiplicity of Infection
qRT-PCR: Quantitative Reverse Transcription PCR
fLuc: Firefly Luciferase
rLuc: Renilla Luciferase
ORF: Open Reading Frame
dpi: Days Post Infection
